# Improving infant Neurocognitive Development and Growth Outcomes with micronutrients (INDiGO): A protocol for an efficacy trial in rural Gambia

**DOI:** 10.12688/wellcomeopenres.21282.1

**Published:** 2024-07-16

**Authors:** Sophie E. Moore, Samantha McCann, Ousman Jarjou, Muhammed A. Danjo, Bakary Sonko, Ebrima Sise, Samuel Beaton, Daniel Tod, Greg Fegan, Andrew M. Prentice

**Affiliations:** 1 Department of Women and Children’s Health, King's College London, London, England, UK; 2 MRC Unit The Gambia at the London School of Hygiene and Tropical Medicine, Fajara, The Gambia; 3 University of Swansea, Swansea Trials Unit, Swansea, UK; 4 Mahidol University, Mahidol Oxford Tropical Medicine Research Unit, Mahidol, Thailand

**Keywords:** micronutrients, pregnancy, lactation, infancy, neurodevelopment, growth, intervention, Gambia

## Abstract

**Background:**

Undernutrition during the early years of life has a harmful and irreversible impact on child growth and cognitive development. Many of the interventions tested to improve outcomes across infancy have had disappointing or inconsistent impact, a common feature being the absence of any attempt to provide nutritional supplements to infants during the first six months. With increasing evidence of micronutrient deficiencies in this age group, alongside strong evidence that growth and developmental deficits begin before six months, a renewed focus on the micronutrient status of infants is required.

**Methods:**

This study is a five-arm, double-blind, placebo-controlled, randomised efficacy trial of micronutrient supplementation to mothers (during pregnancy or pregnancy and lactation) and infants (Day 8 to six months of age) in rural Gambia, where rates of micronutrient deficiencies are high. 600 pregnant women (<20 weeks gestation) will be enrolled into one of five trial arms and followed to 12 months post-partum. The primary outcome will be infant brain development at six months, with micronutrient status, growth and neurocognitive development to 12 months as secondary outcomes.

**Discussion:**

This novel research will identify the most efficacious way of improving micronutrient status in infancy, and assess impact on infant developmental outcomes, providing an evidence base for future effectiveness trials and policy recommendations.

**Trial registration:**

ISRCTN registry ( ISRCTN15063705, 09/07/2021); Pan African Clinical Trials Registry ( PACTR202201552774601, 21/01/2022).

## Plain Language Summary

Deficiencies of micronutrients in infants and young children can have a long-lasting impact on growth and development. This is especially relevant in populations with poor diets. However, little research has focused on interventions to improve micronutrient deficiencies in the first six-months of life, during the period when exclusive breastfeeding is recommended.

Here we describe the protocol for a randomised trial among women and infants living in sub-Saharan Africa, to determine whether supplementation with micronutrients to mothers (in pregnancy, or pregnancy and lactation) or supplementation to infants directly (while promoting exclusive breastfeeding) will improve infant development across the first 12 months of life. This trial will test the effect of supplementation on infant brain development, micronutrient status and growth.

This novel research will determine the best way to improve infant micronutrient status and developmental outcomes, providing important data for policy considerations.

## Introduction

Undernutrition during the early years of life has a harmful and irreversible impact on child development ^1^. This is particularly relevant in low- and middle-income countries (LMIC), where one in three children fail to reach their developmental milestones by school-age ^2^. Early deficits in neurodevelopment can predict poorer mental health, academic achievement and economic productivity across the lifespan ^3^. The first 1000 days of life, the period from conception to two years of age, has been identified as a critical period for both physical and neurocognitive development and emphasis is now placed on understanding pathways to developmental deficits during this time, so that effective interventions can be identified and taken to scale. There remains an unmet need as many of the interventions tested to date have had disappointing or inconsistent impact. These have included trials of combined nutritional supplementation to both mothers and infants (e.g. 4– 8) and, more recently, testing of multiple interventions (e.g. nutrition, water, hygiene and sanitation) introduced simultaneously (reviewed in Humphrey *et al.* ^9^).

A common feature has been the absence of any intervention delivered directly to the infant during the first six-months of life. This is especially the case with nutritional interventions, with the exception of studies that have focused on the promotion of exclusive breastfeeding ^10^. Recent data from The Gambia has highlighted that high rates of micronutrient deficiencies occur before six months of age, even among exclusively breastfed infants ^11^ with growth faltering occurring very early in life (<6 months of age) ^12, 13^. Further, preliminary data from the Brain Imaging for Global Health (BRIGHT) study also indicates that, in comparison to a UK population, neurocognitive development among Gambian infants is impacted across this period ^14^. Micronutrients are critical for brain development during the first months of life ^15^, yet this window of vulnerability is neglected with respect to maternal and child health policies.

For standard antenatal care, WHO policy recommends daily iron-folic acid supplementation alone ^16^, although debate persists regarding the additional use of multiple micronutrient (MMN) supplements to provide other essential micronutrients ^10^. However, with the knowledge that micronutrient stores may become rapidly depleted if supply is not maintained, even supplementation with MMNs to women in pregnancy may not be adequate to ensure sustained maternal or infant micronutrient sufficiency across the post-partum period. Current WHO policy does not include recommendations to supplement women during lactation, and the existing policy on exclusive breastfeeding to six months implies that breastfeeding is sufficient to supply adequate micronutrients over this period despite acknowledgement of a weak evidence base ^17^. Advanced methods for assessment of micronutrient concentrations in breastmilk have highlighted possible deficiencies in critical micronutrients in women with low micronutrient intakes or stores ^18^, supporting the need to improve maternal and/or infant status. Providing a stronger evidence base for linked-up policy across the pregnancy-post-partum continuum would ensure the maintenance of micronutrient supply to young infants, improving development at this critical phase of life.

## Trial objectives

On this backdrop, the primary aim of the INDiGO trial is to determine the most efficacious method of micronutrient supplementation to women and infants from a population with high rates of deficiency, to improve neurocognitive development during early infancy (birth to six months of age).

The secondary objectives are to:

1. Assess the impact of micronutrient interventions during pregnancy, lactation and infancy on maternal and infant micronutrient status.
2. Assess the impact of micronutrient interventions during pregnancy, lactation and infancy on infant growth trajectories across the first 12 months of life.
3. Assess the impact of micronutrient interventions during pregnancy, lactation and infancy on infant morbidity and infant feeding patterns across the first year of life.

## Protocol

### Trial design

**Figure 1.**
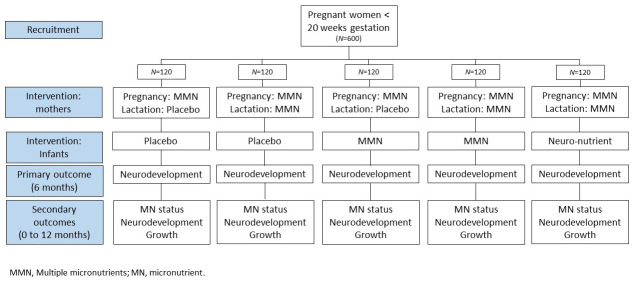
Schematic of Trial Design.

This is a five arm, double blind, placebo controlled, individually randomized efficacy trial comparing combinations of multiple micronutrients to pregnant and lactating women and their newborn infants in rural Gambia. At enrolment, women will be randomised to one of five trial arms ( Figure 1) and women and their infants will remain in the same arm through to six months post-partum. The primary trial outcome will be assessed in infants at six months of age, with further follow up of women and infants until 12 months post-partum.

### Sampling

The INDiGO trial will be based at the Medical Research Council Unit The Gambia at the London School of Hygiene and Tropical Medicine (MRCG) in The Gambia, West Africa. Participants will be recruited from villages within the Kiang regions of The Gambia. These rural communities largely rely on subsistence agriculture and food availability fluctuates widely across the year. The wet season, lasting from July to October, represents a “lean” period because stored staple foods from the previous year’s harvest are nearly depleted and the agricultural workload of much of the community increases. As a consequence, a seasonal variation in birth weight results, which can be improved by supplemental nutrition during gestation ^19^. Despite considerable improvements over the past decades, rates of childhood undernutrition within this community remain high ^20^.

All women (aged 18 to <40 years), resident in the study villages and identified as pregnant (at antenatal clinics or through demographic surveillance), will be invited to participate in the study. Following informed consent, women will then be invited for an ultrasound examination by the study midwife at the MRC Keneba field station clinic at the MRCG Keneba. Eligible women will then be enrolled into the trial and randomized to one of the trial arms. The trial will enrol up to 138 women per trial arm (690 total) with the aim of having data from 120 infants per trial arm (600 total) at the timepoint of the primary outcome.

### Informed consent

Individual consent for the study will be sought, with pregnant women providing consent for both herself and her dependent infant. Field assistants will be trained to explain the full details of the trial to all eligible women, in English or a local language they understand (Mandinka, Fula or Wolof, see eligibility criteria, below), covering all aspects of the study as laid out in the ‘Participant Information Sheet’ (PIS). Literate women will then be given a printed copy of the PIS to read and refer back to. In cases of illiterate women, the entire consenting process will be done in the presence of an impartial literate witness. Any questions that arise will be answered by the field assistants and one of the study investigators will also be available for further clarifications and explanations if required. Participation in the trial is entirely voluntary, and where women are willing to be involved, Informed Consent will be obtained in writing or via thumb print.

### Eligibility criteria

Eligibility criteria are: Pregnant women with an ultrasound confirmed viable singleton pregnancy of < 20 weeks (± 7 days) gestation. Women should be willing to take a trial product (capsule) daily from 20 weeks of pregnancy until six months post-partum. They must also be willing for their infant to receive a daily trial product (syrup) from Day 8 until 6 months of age. Women must intend to remain resident in the study area for the duration of the trial (to the point the infant is aged 12 months) and they must be considered in a position to provide their own full, informed consent to participate.

Additional exclusion criteria during the pregnancy phase of the study will include:

- Severe anaemia (<7g/dL)
- Any known history or evidence of chronic disease (including HIV, TB, non-pregnancy-induced-hypertension or diabetes). Women will be offered HIV Voluntary Counselling and Testing (as part of routine antenatal care) and, where positive, excluded from the trial
- If the primary language of the mother is not Mandinka, Fula or Wolof

Exclusion criteria during the post-partum / infancy phase of the study will include:

- Preterm infants (< 32 weeks gestation at delivery)
- Very low birth weight infants (<1.5kg at delivery)
- Infants identified at any follow-up point as having severe-acute malnutrition (weight-for-height z score of <-3SD)
- Non-breastfeeding mother-infant pairs
- Unwilling to avoid the ingestion of – or for their infant to avoid the ingestion of – other micronutrient supplements during the study period
- Any condition of the mother or infant that, in the opinion of the investigator, might compromise the safety or well-being of the participant or compromise adherence to protocol procedures (including the identification of severe neurodevelopmental conditions, such as cerebral palsy).

### Study procedures

Screening: Potential study participants will be identified through antenatal clinics and demographic records. During the screening visit, mothers will have the full purpose of the study explained to them and asked for their written informed consent. These mothers will be invited to have an ultrasound examination for the confirmation and dating of their pregnancy. Women whose primary language is Mandinka, Fula or Wolof and with a singleton pregnancy, <20 weeks gestation will be invited back for a further enrolment (baseline) visit at the point they reach 20 weeks gestation.

Enrolment (baseline): At 20 weeks (±7 days) gestation, all women who passed the initial screening will be invited to the MRC Keneba clinic for an enrolment assessment. At this visit, each woman will receive a further ultrasound examination where fetal biometry will be measured and recorded, and a standard antenatal care examination (blood pressure, haemoglobin assessment and urinary analysis) performed. Maternal anthropometry (weight, height, head circumference, mid-upper arm circumference) will also be measured and a blood and urine sample collected and stored. If confirmed eligible by a member of the clinical team, antenatal supplementation will commence after this visit according to their allocated randomisation arm.

Pregnancy phase: Following the week 20 clinic visit, women will be visited daily for observed supplementation (one capsule per participant per day). Once a week, data on maternal morbidity will be collected by questionnaire. Women will then be invited for further antenatal clinic visits at 28 and 36 weeks’ gestation. The same assessments as performed at the 20 week visit will be conducted and recorded.

Delivery and neonatal examination: A system of village assistants will be put in place (one village assistant per study village) to enable early notification to the study team as soon as an enrolled woman goes into labour. Following delivery, study field assistants will aim to collect a sample of cord blood and transport it to the MRCG Keneba laboratory. The study midwife or clinical delegate will aim to visit the mother and neonate within 72 hours of delivery for a maternal and neonatal health examination and the measurement of neonatal anthropometry (weight, length, head circumference, mid-upper arm circumference). In recognition that some women may travel outside of the region to deliver, visits that occur up to 10 days post-partum will still be accepted within the protocol. An eligibility check to proceed into the post-partum supplementation phase will be conducted at this point (see eligibility criteria, above).

Post-partum/infancy phase: From Day 8 until 6 months post-partum, mother-infant pairs will be seen daily for supplement administration. For women, supplementation will be observed (by field assistants) and for infants, field assistants will administer the syrup directly. Only where 5 or more consecutive daily visits are missed will this constitute a protocol deviation/non-compliance.

From 1 week to 7 months of infant age, data will be collected weekly by questionnaire on maternal and infant morbidity and infant feeding practices. From 7 months (e.g. one month following the cessation of supplementation) to 12 months of infant age, these assessments will follow a monthly schedule. 

At monthly home visits until 12 months post-partum, infant anthropometry will be recorded, and stool samples collected (for the visits at 1, 6 and 12 months post-partum, these assessments will take place as part of clinic visits, rather than at home – see below). When the infant is three months of age, the mother will be asked to provide a 10mL sample of breast milk. At the home visit when the infant is 6 and 12 months of age, we will conduct a Home Observation Measure of the Environment (HOME) assessment, which includes 90 minute observation of the infant’s ‘normal’ home life including recording details on the organization of the home environment, parental involvement in care, variety of daily stimulation and appropriate play materials ^21^.

At 1, 6 and 12 months post-partum, mother-infant pairs will be invited to MRC Keneba for a study visit. At all visits, maternal and infant anthropometry will be measured and a 10mL sample of breast milk will be collected from mothers. When the infant is one month of age, a finger prick blood sample will be collected from each infant and a 7.5mL sample of venous blood from mothers. At 6 and 12 months, further samples of maternal (7.5mL) and infant blood (5mL) will be collected. All samples will be processed and biobanked for subsequent analysis (see Laboratory evaluations section).

Infant neurocognitive development will be evaluated at each of these clinic visits.

Assessment of infant neurocognitive development: This study will utilize a set of complementary tools to measure neurocognitive development from 1-12 months of age.

The Mullen Scales of Early Learning (MSEL) is a behavioral assessment suitable for use from birth to 68 months of age. The MSEL consists of five scales (gross motor, fine motor, visual reception, receptive language, expressive language) was previously adapted for this population and implemented in the BRIGHT study ^22^. Each scale is made up of increasingly complex tasks and scoring is based on the completion of each task ^22^. The MSEL will provide a broad measure of cognitive and motor development. At 6 and 12 months of infant age, the MSEL will be complemented by an Infant Behaviour Questionnaire (IBQ) to collect additional information on infant attention.

In addition, eye tracking technology (Tobii Pro Fusion 120Hz, Tobii AB, Sweden) and functional near infra-red spectroscopy will be used to objectively measure visual and auditory attention, respectively. Attention is the foundation for learning and among the most rapidly developing aspects of cognition in the first 6 months of life ^23^ and may, therefore be particularly vulnerable to nutritional deficiencies within this period.

Eye tracking: A battery of eye tracking assessments will be used, previously implemented as part of the BRIGHT study in The Gambia ^24^. These assessments will include measures of social attention, working memory, attentional flexibility, habituation and novelty detection which are engaging and require only passive interaction from infants.

fNIRS: We will implement an fNIRS assessment of auditory habituation and novelty detection, previously implemented in BRIGHT and as fully described in Lloyd-Fox *et al.* 2019 ^14^. The protocol will remain largely the same with the exception of the system used. Within this trial, fNIRS will be conducted using the LUMO system (Gowerlabs, UK) ^25^. This system reflects recent engineering advances within the field and has been reported to offer superior data quality and participant retention ^25^.

Finally, we will use the same fNIRS system (LUMO, Gowerlabs, London) to assess functional connectivity while the brain is at rest. Functional connectivity, or functional network organization, is a measure of synchroneity in the firing of neurons in different brain regions. Functional connectivity has previously been associated with adversity in early life ^26, 27^ and later developmental outcomes ^27^.

The clinic visit at 1 month of age will serve as a pseudo-baseline for developmental trajectories, the 6 months visit will be the primary endline and 12 month visit the secondary endline. All assessments described above will be completed at each clinic visit with the exception of eye tracking at 1 month as it is not feasible to conduct this assessment in this age group.

Final study visit: The final study visit will be conducted when the infant reaches 12 months of age. As well as the developmental assessments outlined above, maternal and infant anthropometry, maternal and infant blood samples, and a breast milk sample will be collected, as described in the post-partum/infancy section above and in more detail below.

### Laboratory evaluations

Details of sample collection protocols and laboratory evaluations are as follows:

Mothers: Blood samples collected at 20, 28 and 36 weeks of pregnancy and at 1 and 6 months post-partum will be processed (including initial assessment of haemoglobin levels). Plasma from these samples will be frozen and biobanked for subsequent analysis. Planned assessments include the measurement of nutritional biomarkers including iron and inflammation status (to be performed at MRCG Keneba), B12 and choline (to be shipped to the US for analysis at the Western Human Nutrition Research Center, Davis, California). At these visits, a spot urine sample will also be collected from each participating mother for urine analysis (dipstick), as part of routine antenatal care. Frozen urine aliquots will then be stored for shipment to Switzerland for analysis of iodine status biomarkers at the Nutrition Research Unit, University Children's Hospital Zurich. At 1, 3, 6 and 12 months post-partum, women will be asked to hand-express a sample of breast milk (up to 10mL). These samples will be separated into smaller aliquots and frozen for subsequent shipment to partner laboratories in the US and Switzerland for analysis of nutritional biomarkers.

Infants: Blood samples (cord blood, fingerprick at one-month and 5mL at six- and 12-months post-partum) will be processed and samples of plasma frozen and stored for subsequent analysis. The same biomarkers as indicated for mothers’ samples (above) will also be assessed in infant samples. Infant thyroglobulin will be measured in infant blood samples as an indicator of iodine status, as urinary iodine levels are not a robust indicator of infant status. Infant stool samples will be processed into smaller aliquots and biobanked for future investigation into the infant microbiome.

### Randomization and blinding

At the point of enrolment in pregnancy, mothers will be randomized to 1 of the 5 study arms, using block randomization, according to a computer-generated randomization scheme. Mothers and their infants will remain in the same group for the duration of the trial. A randomization code will be produced using the STATA software, and imported into REDCap by the database manager, with support from the trial statistician.

### Interventions

During pregnancy and lactation, women will be randomized to receive a daily MMN supplement (capsule) or placebo. For the MMN intervention arms, we will use the UNIMMAP formulation, a preparation of 15 micronutrients specifically designed for pregnancy and as formulated by UNICEF/WHO/UNU ^28^. A single capsule provides the Recommended Dietary Allowance (RDA) for each micronutrient ( Table 1). Placebo capsules (maltodextrin) will look and taste identical.

**Table 1.** INDiGO Micronutrient Intervention Products Composition.

Micronutrients	Dose/day		
	UNIMMAP	Infant MMN	Infant neuro-nutrient
Vitamin A (ug RE)	800	175	350
Vitamin D (IU)	200	200	400
Vitamin E (mg)	10	2	4
Thiamine (mg)	1.4	0.2	0.4
Riboflavin (mg)	1.4	0.25	0.5
Niacin (mg)	18	3	6
Folic acid (µg)	400	50	100
Vitamin B6 (mg)	1.9	0.25	0.5
Vitamin B12 (µg)	2.6	0.4	0.8
Vitamin C (mg)	70	12	24
Zinc (mg)	15	1.5	3
Iron (mg)	30	2.2	4.4
Iodine (µg)	150	45	90
Selenium (µg)	65	10	20
Copper (mg)	2	0.3	0.6
Choline (mg)			125

For the MMN intervention arms during pregnancy and lactation, we will use the UNIMMAP formulation ^28^. For infants in the MMN intervention arm, we will use the same 15 micronutrients as in UNIMMAP, but at levels appropriate for this age group ^31^. For the neuro-nutrient arm, we will use the same preparation as the infant MMN arms, but at twice the dose and with the addition of choline (a nutrient essential for infant brain development known to be insufficient in this population) ^32^. These levels are below the maximum level for young infants, according to both EFSA ^33^ and the Codex Alimentarius of the FAO ^31^.

All women will receive the UNIMMAP supplement during pregnancy and randomisation to the intervention (UNIMMAP) or placebo capsule will start post-delivery. To date, the UNIMMAP formulation has been used in multiple clinical trials globally and has been shown to offer similar benefits (in prevention of iron-deficiency anaemia) or out-perform (for several birth outcomes) ^29, 30^ iron folic acid. The most recent update to the WHO recommendations on antenatal care for a positive pregnancy experience states that antenatal multiple micronutrient supplements that include iron and folic acid are recommended in the context of rigorous research, where research in this context includes controlled clinical trials ^16^. In line with WHO recommendations, and in view of the context of the trial (e.g. rigorous research) we see benefit in providing all women enrolled into the study with multiple micronutrients, instead of iron folic acid.

As indicated, from delivery until six-months post-partum women will be randomized to receive this same UNIMMAP preparation as used in the pregnancy phase (test) or a maltodextrin preparation (control). Current policy does not include provision of iron folic acid or multiple micronutrients through the period of lactation. However, given the additional nutritional demands that lactation puts on a woman, we will test whether extending the supplementation period through the recommended period of exclusive breastfeeding confers additional health benefits to both mother and infant. A number of recent trials have included the UNIMMAP formulation in lactating women, with no adverse effects identified (e.g. 5). We do not therefore, foresee any risks of the proposed intervention during lactation.

From birth until six months of age, infants will receive a daily supplement of MMNs or placebo. To enable direct comparison between route of supplementation (trial Arms 1-4), the MMN formulation will be a combination of the same 15 micronutrients given to women during pregnancy and lactation, but at levels appropriate for this age group ( Table 1). Arms 3 and 4 of the trial will receive a ‘basic’ supplement; Arm 5 will receive a formulation identical in composition to the basic infant MMN formulation, but with twice the dose and with the addition of choline (a nutrient essential for infant brain development known to be insufficient in this population). Inclusion of this fifth ‘neuro-nutrient’ (NN) comparative arm will test the third research hypothesis, i.e. that a tailored neuro-nutrient MMN supplement targeted to infants will out-perform the standard formulation of MMN with respect to infant brain development. The formulation of the supplement is guided by the reported nutrient requirements of early brain development ^1^ and has been developed in accordance with dietary reference values for young infants ^33, 31^. Infant supplements (intervention products and placebo) will be provided as a syrup and dropped directly into the infants mouth. Supplementation will commence in the second week of life, after the infant has been named (Day 7 in The Gambia; prior to this the mother-infant pair have a period of confinement for breastfeeding to establish).

The trial design acknowledges that indirect supplementation (e.g. supplementation to the mother during lactation) has little impact on a number of critical micronutrients in milk (e.g. iron) and that direct supplementation (i.e. to the infants themselves) may be the most effective route. As this trial is designed to assess efficacy, daily supplementation will be observed (for mothers) or administered directly (for infants) by a member of the field team.

### Data quality and standards

Most data will be collected into electronic case report forms (eCRFs) via a trial-specific, dedicated REDCap database. Other data types, such as those collected directly from the fNIRS/eye tracking systems, will be uploaded daily into the server. Data from both sources will be linked using the participant ID number, which will be a unique number allocated at consent into the trial.

eCRFs will be accessed via benchtop computers on the MRC Keneba camp or tablets in the field / at the site of data collection. Where there is internet connectivity, collected data will be stored directly into the central server at MRCG Keneba. Where there is no/unreliable internet connectivity, data will be stored locally on the tablet and later (within 24 hours of collection) synchronized to the server.

Before the start of the trial, all project staff will be trained on study data collection and handling, and training documented. Only staff who have been trained will be able to access and complete study eCRFs. To ensure standardization of processes, standard operating procedures (SOPs) and Study Specific Procedures (SSPs) will be used, and principles of Good Clinical Practice will be adhered to throughout. To help ensure data quality, data entry screens will be designed with range checks, skip patterns and validations, where appropriate. Mandatory field checks and other computable data (e.g., dates of visit) will be prepopulated to eliminate errors. The study Data Manager will conduct regular data cleaning routines to flag data queries that were not picked up at earlier stages. All data queries will be answered in writing by a senior member of the study team involved in the generation of the data (e.g. clinical team, lab team, field team, or neurodevelopment team) normally within one week.

### Sample size and statistical analyses

We used published data on the fNIRS Habituation and Novelty Detection (HaND) task from 150 Gambian infants enrolled into the BRIGHT study at five months of age for the purpose of determining the required sample size ^14^.

With a mean (standard deviation, SD) of 0.06(0.51) mM, 120 mother-infant pairs per arm have 95% power to detect a 0.5 SD difference in response at P<0.05. We used a penalised alpha (0.015) in our design to allow for multiple comparisons at the 0.05 threshold for analysis. A 15% attrition to 12 months post-partum requires 138 women per arm to be enrolled in pregnancy.

The primary analysis on the fNIRS habituation will be by arm allocated using least squares multiple regression models. Per protocol analyses will further investigate the potential effectiveness of the interventions. Adjustments for factors unbalanced across arms at baseline will be made. We will explore the combination (interaction) of treatment effects but haven’t powered the study on this. However, given our ability to look at three comparisons, we believe this is a reasonable compromise. Formally detecting a 2x2 interaction effect requires at least 4 times the sample size for a single factor whereas we are suggesting a 3-fold inflation for three separate tests i.e. Arm 1 versus Arm 2, Arm 3 versus Arm 4 and Arm 4 versus Arm 5 (the latter being powered as a one-sided superiority test).

For secondary outcomes, we will look to examine maternal and infant micronutrient status through regression techniques. If the data is markedly not normal, appropriate non-parametric tests (T-test or ANOVA) will be employed.

For each of the four anthropometric measures (length, weight, head circumference and MUAC), both z scores and raw measurements will be analysed using mixed linear regression techniques and we will also group this data into categories (e.g. stunted vs. not stunted, wasted vs. not wasted, undernourished vs. not undernourished) and analyse these outcomes using generalised binomial regression to account for multiple measures.

The number of morbidity episodes and number of participants having morbidity episodes will be calculated at set time points. The number of episodes will be compared by arm using an appropriate count model, either Poisson or negative binomial or other such related.

The infant’s age at cessation of exclusive breastfeeding will be determined from the questionnaire and be visualised by a Kaplan-Meier. Additionally, an exclusive breastfeeding (Y/N) categorical variable will be derived at set time points and analysed using Chi-squared test.

Age adjusted T scores will be calculated for MSEL assessments at each time point. Both raw scores and T scores will be analysed using multiple linear regression techniques. We will also group this data into categories (e.g. by quartile) and analyse using logistic regression. Eye tracking measures of visual attention are continuous variables and will be analysed using multiple linear regression techniques. For functional connectivity data, t tests will be performed using the R value of Fisher z transformed connectivity matrices.

The full statistical analysis plan (SAP) is available on Open Science Framework ( https://osf.io/nksv3/).

### Data and safety monitoring

The design of this efficacy trial necessitates daily contact between trial staff and study participants during the active phase of the trial (20 weeks of gestation through to six-months post-partum), weekly to seven months post-partum, and monthly thereafter, with frequent collection of morbidity reports. This close surveillance will help ensure prompt treatment or onward referral as relevant.

The trial will be overseen by a Data Safety Monitoring Board (DSMB) who will review relevant safety data at pre-agreed time intervals. The DSMB will be provided with summary data during open sessions with the trial management team and safety data will further be tabulated by coded treatment arm during a closed session with the DSMB alone. Where the DSMB feels there is ‘potential for harm’, unblinding of the treatment arms can be requested. The DSMB will additionally monitor rates of recruitment and retention and flag any relevant concerns to the trial team and Sponsor.

In addition to the DSMB, a Local Safety Monitor (LSM) has been appointed. This is a clinician, employed by the MRCG, but independent to the trial team. The LSM will review all serious adverse events as they occur, and regularly review adverse events, with a particular focus on causality, trends in loss to follow up, or any issues that are considered as immediate safety concerns. The LSM will report to the trial PI and/or the DSMB as relevant.

### Withdrawal of participants

In cases where participants chose to withdraw from the trial, details will be entered into the database regarding the reason for withdrawal. In cases where adverse events remain unresolved, participants will be followed up by a member of the trial’s clinical team. In cases of withdrawal, any data and samples already collected to the point of withdrawal will be used.

### Participant confidentiality

In accordance with the UK General Data Protection Regulation (UK GDPR) and the Data Protection Act 2018, any identifiable data collected will be stored securely and the confidentiality of participants will be protected. Data sharing will follow relevant regulatory protocols, including – where possible – only sharing fully deidentified data.

### Future use of stored biological samples and data

Aliquots of all biological samples collected (blood, urine, breast milk, stool) will be biobanked at -80 ^o^C for future analyses, including during export to collaborating institutes overseas for analysis. The informed consent process incorporates permission from participants for long-term storage, shipment and use of samples. Any future use of samples, and accompanying metadata, will require the relevant institutional approvals, and can be channelled through the trial PI (SEM).

### Ethics

The trial has been approved by the Research Ethics Committee at King’s College London (project reference HR/DP-20/21-23914, 12 ^th^ March 2021) and by the MRCG Scientific Coordinating Committee and the joint Gambia Government / MRCG Ethics Committee and by the Ethics Committee at LSHTM (project reference 25071, 12 ^th^ May 2021). All subsequent amendments will go through the same process with changes communicated, as relevant, to investigators, trial participants, trial registries and relevant regulatory bodies. The trial will be conducted in accordance with the principles defined in the ICH Harmonised Tripartite Guideline for Good Clinical Practice and the Declaration of Helsinki in its current version.

### Dissemination and data access

All key findings from this study will be submitted for publication in Open Access peer-reviewed journals and presented at relevant international conferences. We will also disseminate all relevant findings to Gambian and international stakeholders and organizations.

Any requests for use of study data will go through approval from the trial Sponsor ( www.khpcto.co.uk) and Ethics Committee at MRCG ( ethics@mrc.gm). Requests must be in agreement with data sharing policies at both institutes. Only anonymised data will be shared.

### Study status

The first participant was randomised in May 2023. It is anticipated that all women will be enrolled by September 2025 and follow up to the final infant reaching 12 months of age will be completed by January 2027.

## Discussion and conclusion

Undernutrition during the early years of life has a harmful and irreversible impact on child growth and development. Many of the nutrition-specific and nutrition-sensitive interventions tested to improve outcomes across infancy have had disappointing or inconsistent impact. A common feature of these interventions, however, is the absence of nutritional support to infants during the first six months, beyond the promotion of exclusive breastfeeding. With increasing evidence that growth and developmental deficits begin before six months, alongside evidence of micronutrient deficiencies in this age group, there is a renewed focus on interventions to support infants both directly and indirectly (via their mother) during this time.

The INDiGO trial will directly address this knowledge gap and aims to identify both the optimal route (maternal during pregnancy or pregnancy and lactation or combined maternal and infant interventions) and formulation for improved infant developmental and growth outcomes. The INDiGO trial will not be without challenge; some may see this as questioning WHO policy on exclusive breastfeeding. This is not the case, rather, it is reflecting the lack of evidence on the adequacy of data on micronutrient quality in breastmilk at the time WHO policy was set, and a response to the growing evidence that the first six months represent a critical period of nutritional vulnerability for young infants born to women with insufficient micronutrient intakes, even when breastfed. If this trial demonstrates benefit of supplementation (to mothers or infants or both) across the first six months, this will not only support current policy but provide evidence that greater efforts must be made to optimize micronutrient status in women of reproductive age, and support women and infants in contexts where micronutrient deficiencies are common.

## Data Availability

No data are associated with this article. On completion of the trial, data will be made available via Open Science Framework ( https://doi.org/10.17605/OSF.IO/NKSV3). Open Science Framework: Improving Infant Neurocognitive Development and Growth Outcomes with Micronutrients (INDiGO): A protocol for an efficacy trial in rural Gambia. https://doi.org/10.17605/OSF.IO/NKSV3 ^34^ This project contains the following extended data: INDiGO_ICD_v3.0.pdf INDiGO_SAP_v1.0_signed.pdf INDiGO_SPIRIT_Checklist_180624.pdf INDiGO_TrialProtocol_v6.0.pdf Data are available under the terms of the Creative Commons Zero "No rights reserved" data waiver (CC0 1.0 Public domain dedication).
