# Supplementary material for: Improving infant Neurocognitive Development and Growth Outcomes with micronutrients (INDiGO): A protocol for an efficacy trial in rural Gambia
Source: Wellcome Open Res. 2024 Jul 16;9:377. [Version 1] doi: 10.12688/wellcomeopenres.21282.1 (PMC11474154; doi:10.12688/wellcomeopenres.21282.1)
Supplement: Peer review history

# Reviewer report: Reviewer response for version 1

**DOI:** 10.21956/wellcomeopenres.23538.r97181

**Published:** 2024-10-14

**Authors:** Laeticia Celine Toe

**Affiliations:** 1 Unité Nutrition et Maladies Métaboliques, Institut de Recherche en Sciences de la Santé (IRSS), Bobo-Dioulasso, Burkina Faso; 2 Ghent University, Ghent, Belgium

Moore et al. will be conducting a five-arm, double-blind, individually randomized, placebo-controlled clinical trial to investigate the most efficacious method of micronutrient supplementation during pregnancy, lactation, and early infancy in rural Gambia. The study aims to improve neurocognitive development and growth outcomes in infants.

 The trial will enroll 690 pregnant women, expecting to retain 600 infants for the primary outcome assessment. Participant pregnant women will be randomized at inclusion for both prenatal and postnatal supplementation which encompasses their infant receiving infant supplementation, with a placebo group for comparison.

 The study has a strong design with directly observed intake of supplements, which is likely to guarantee a high degree of compliance. The study is well described with clear strategies for recruitment, retention, and management of potential loss to follow-up are described.

 The primary outcome is infant neurocognitive development at six months, while secondary outcomes include maternal and infant micronutrient status, growth trajectories, and morbidity during the first year of life. The primary outcome will be measured through a combination of behavioral assessments and neuroimaging techniques. The use of neurocognitive assessments (MSEL, eye tracking, fNIRS) in this population is a strength, allowing for detailed and objective measurements of infant development.

 The protocol acknowledges the potential benefits of supplementation and minimal risks, supported by previous trials. The close monitoring of participants and daily interactions help mitigate potential risks.

 Overall, this is a well-designed study addressing a significant knowledge gap in maternal and child nutrition. However, revisions should address the slight inconsistencies in the sample size calculation, further clarify the randomization process, and improve the descriptions of primary outcomes and statistical methods.

***Study objectives and hypothesis:*** While the objectives of the study are clearly stated, the (testable) hypothesis could only be inferred from the sample size calculation. The reader could benefit more from an explicitly stated hypothesis next to the objectives.

***Participant selection:*** The justification for excluding women based on language should be carefully reconsidered or nuanced to avoid discrimination. To the best of my knowledge, in African Countries people can speak languages that are not necessarily their primary language. Reading between the lines, I could understand that language assessments in infants might be the reason for such selection criteria. I recommend the authors state that clearly if indeed that was the reason.

***Randomization*** *:* The study uses block randomization with five trial arms. However, the block sizes for randomization are not specified, nor is there mention of whether block sizes will vary. This information should be included for transparency.

***Blinding*** *:* The paper could benefit of more details on how allocation concealment and blinding will be ensured for both participants and investigators. This should be clearly explained for reproducibility.

***Intervention*** *:* Justifications for including choline as a key component of the neuro-nutrient arm could be strengthened with additional literature references on its role and its deficiency, especially in the Gambian population or in LMICs in general​.

***Baseline patient characteristics: *** As pre-pregnancy BMI may not be available in this study, I recommend that the authors also add mid-upper arm circumference 5MUAC) to the anthropometric measurements at baseline.

***Biological samples: *** Mothers will be asked to provide hand-expressed breast milk samples at 1, 3, 6 and 12 months post-partum. “ *These samples will be separated into smaller aliquots and frozen for subsequent shipment to partner laboratories in the US and Switzerland for analysis of nutritional biomarkers.”* As foremilk and hindmilk have typically different nutritional composition ( **https://doi.org/10.1002/fbe2.12049** , **https://doi.org/10.3390/nu10121828** ), I recommend that the authors look into full expression of 1 breast, homogenize and then aliquot. The remaining milk (if any) can be fed back to the baby using a cup (to avoid nipple confusion).

 The authors state that ‘ *Infant stool samples will be processed into smaller aliquots and biobanked for future investigation into the infant microbiome.”* However, no objective (primary or secondary) links directly to microbiome investigations. Can the authors clarify/ provide a research question that would be addressed by the microbiome investigation?

***Sample size calculation and statistical analysis:*** The sample size justification appears to have some inconsistencies. The protocol mentions that the sample size is powered for 12-month outcomes, even though the primary outcome is measured at six months. The authors should revisit the sample size calculation to ensure clarity. The statistical methods are generally appropriate but require some refinement. For example, the description of parametric and non-parametric tests should be corrected as T-tests and ANOVA are parametric, not non-parametric tests​.  Also, it would be helpful to clarify which groups will be compared for primary and secondary outcomes.

***Strengths and Limitations: *** One limitation is that the study relies solely on the placebo-controlled design to assess the primary outcome, which may overlook subtle yet clinically significant effects of low-dose supplementation. The discussion would benefit from a deeper acknowledgment of potential weaknesses, such as the possibility of overlooking small effect sizes (<0.5 SD)​. Although the study procedures include a directly observed/ administered supplement intake, there is a possibility of different levels of compliance. It would be then worthwhile examining a dose-response relationship in the statistical analysis.

Is the study design appropriate for the research question?

Yes

Is the rationale for, and objectives of, the study clearly described?

Yes

Are sufficient details of the methods provided to allow replication by others?

Partly

Are the datasets clearly presented in a useable and accessible format?

Not applicable

Reviewer Expertise:

Nutrition, Gut microbiota and maternal and child health

I confirm that I have read this submission and believe that I have an appropriate level of expertise to confirm that it is of an acceptable scientific standard.

## References

[1] : Differences in the content of human milk oligosaccharides between foremilk and hind milk. https://doi.org/10.1002/fbe2.12049 10.1002/fbe2.12049

[2] : Short Communication: Differences in Levels of Free Amino Acids and Total Protein in Human Foremilk and Hindmilk. https://doi.org/10.3390/nu10121828 10.3390/nu10121828 PMC631564030486227

# Reviewer report: Reviewer response for version 1

**DOI:** 10.21956/wellcomeopenres.23538.r91005

**Published:** 2024-08-19

**Authors:** Tor A Strand

**Affiliations:** 1 Department of Research, Innlandet Hospital Trust, Centre for International Health, University of Bergen, Bergen, Norway

This is an important and well-designed study with a clear aim and an easy-to-follow protocol. However, after reading the manuscript, I am still uncertain about the primary objective and outcomes. Therefore, I suggest dedicating a paragraph that explicitly lists and clearly defines the primary and secondary objectives and outcomes.

**I also offer the following suggestions to improve the manuscript:**

**Intervention:**

               • In the statement, “Arm 5 will receive a formulation identical in composition to the basic infant MMN formulation, but with twice the dose and with the addition of choline (a nutrient essential for infant brain development known to be insufficient in this population),” the references supporting the claims about Choline’s essential role and its insufficiency in this population should be provided.

               • Additionally, it would be beneficial to include a justification for why Choline supplementation is only provided during infancy and not during pregnancy.

               • The argument regarding the importance of the “first 1000 days” should be connected to a reference to strengthen the narrative.

**Justification of the Design:**

               • Reference 11 appears incomplete, and its relevance to the argument in the introduction is unclear. Please review and ensure that it adequately supports the claims made.

               • The introduction effectively justifies examining the impact of micronutrients on brain development in early life. However, it could be strengthened by more explicitly justifying the study design, particularly the focus on Choline and vitamin B12 in the “neuro-nutrient” arm. While these nutrients are indeed crucial for brain development, there are many others, and a brief discussion on the rationale for selecting these specific nutrients would be helpful.

               • The overall aim “to determine the most efficacious method of micronutrient supplementation to women and infants from a population with high rates of deficiency” could be perceived as overly ambitious. I suggest narrowing down this aim or ensuring that the objectives are clearly defined and aligned with the aim.

               • The protocol does not explicitly list a primary objective. Furthermore, while secondary objectives are mentioned, they could be more specifically formulated.

               • Regarding the decision to double the dose of vitamins, I suggest providing a justification for this specific increase. For instance, why was a doubling chosen rather than a 50% increase or a tripling dose?

**Inclusion Criteria:**

               • The timing of mother enrollment (week of pregnancy) should be justified. It would also be helpful to explain the rationale behind the second randomization exclusion criteria and how this will be managed in the analysis.

**Randomization:**

               • The block sizes for randomization should be specified, along with whether these sizes will be variable.

**Blinding:**

               • There is no information on how allocation concealment and blinding will be ensured.

**Follow-up:**

               • Please specify whether a dose will be repeated in cases of vomiting or regurgitation.

               • The manuscript includes separate paragraphs for the Tobii device and Eye-Tracking methods, although the Tobii device is used for Eye-Tracking. Consider combining these sections for clarity.

**Laboratory Assessments:**

               • The sentence “Mothers: Blood samples collected at 20, 28 and 36 weeks of pregnancy and at 1 and 6 months post-partum will be processed (including initial assessment of haemoglobin levels)” seems incomplete. Please review and complete the sentence to provide full clarity on the laboratory assessments.

**Primary Outcome Assessment:**

               • While the primary outcome is stated as neurodevelopment at 6 months, the manuscript lacks a concise description of this outcome, including how it will be measured. Although this information can be inferred from the entire manuscript, it would be more reader-friendly to provide it in a distinct, dedicated paragraph.

**Justification of the Sample Size:**

               • The sample size justification needs to be revisited. The statement, “.5 SD effect size 95% power and 15 attrition: adjusted p-value,” suggests that 138 women per arm should be enrolled to account for a 15% attrition rate by 12 months post-partum, expecting 120 children to be available at 12 months. However, the primary endpoints are measured at 6 months. Additionally, the calculation of the attrition rate seems incorrect, as 85% of 690 is not 600. I recommend recalculating the sample size using the assumptions listed to ensure accuracy.A limitation of the study design is that small, relevant effect sizes (less than 0.5 SDs) may be overlooked. I suggest that the authors discuss this shortcoming.

**Statistical Analysis:**

               • It would be helpful to clarify which groups will be compared for the primary outcomes. Given that this is a 5-arm trial, there are multiple ways to conduct these analyses, which should be explicitly stated.

               • While the primary outcomes are assessed at 6 months, the sample sizes appear to have been estimated to ensure sufficient power at 12 months. Please review this discrepancy.

               • The sentence “Per protocol analyses will further investigate the potential effectiveness of the interventions” is somewhat hard to comprehend and could benefit from rephrasing for clarity. Additionally, the sentence “For secondary outcomes, we will look to examine maternal and infant micronutrient status through regression techniques. If the data is markedly not normal, appropriate non-parametric tests (T-test or ANOVA) will be employed” contains inaccuracies. T-tests and ANOVA are parametric tests, not non-parametric. Please correct and update this text accordingly.

               • The sections discussing mixed and generalized models should be reviewed for clarity and completeness.

               • Lastly, including a Kaplan-Meier plot for the cessation of exclusive breastfeeding is puzzling, as this outcome is not listed. If this analysis is essential, it should be explicitly mentioned in the outcomes section.

 Thank you for the opportunity to review this protocol. This is a significant study, and I look forward to seeing the results.

Is the study design appropriate for the research question?

Yes

Is the rationale for, and objectives of, the study clearly described?

Yes

Are sufficient details of the methods provided to allow replication by others?

Yes

Are the datasets clearly presented in a useable and accessible format?

Not applicable

Reviewer Expertise:

Epidemiology, pediatrics, public health, clinical trials in low and middle income countries, micronutrients

I confirm that I have read this submission and believe that I have an appropriate level of expertise to confirm that it is of an acceptable scientific standard.

## References

[1] : Association between Maternal Choline, Fetal Brain Development, and Child Neurocognition: Systematic Review and Meta-Analysis of Human Studies. *Adv Nutr* .2022;13(6) : 10.1093/advances/nmac082 2445-2457 10.1093/advances/nmac082 36041182 PMC9776654
